# Supplementary material for: Effect of long-term use of treated wastewater and clean water mixtures on soil heavy metal accumulation: An assessment using pollution indices
Source: PLoS One. 2025 Dec 4;20(12):e0337424. doi: 10.1371/journal.pone.0337424 (PMC12677520; doi:10.1371/journal.pone.0337424)
Supplement: Attachment — Submitted filename: Plos-one-Response to Reviewers.docx [file pone.0337424.s003.docx]

Dear Editor,

First of all, I would like to thank you and the reviewers for taking the time to evaluate our manuscript. We appreciate the constructive feedback and insightful comments provided, which have significantly helped us improve the quality of our work.

Below, we have provided detailed, point-by-point responses to the reviewers' comments and have made the necessary revisions to the manuscript as follows:

**Reviewer**

1- **Comment:** The pot-based experimental design does not fully replicate field-scale hydrological and biological processes. This limitation is acknowledged, but it should be emphasized more clearly in the Discussion and Conclusion sections.

**Response:** We appreciate the reviewer’s insightful comment regarding the limitations of the pot-based experimental design. In response, we have revised the Discussion and Conclusion sections to more explicitly emphasize that while the pot-based approach allowed for consistent and controlled irrigation treatments over multiple years, it may not fully replicate field-scale hydrological and biological processes such as deep percolation, lateral water movement, or soil-plant interactions.

Accordingly, we now stress that the results should be interpreted with caution when extrapolating to real agricultural settings and have recommended that future studies employ field-scale designs to validate and expand upon our findings.

The following sentences has been added to the Discussion and Conclusion section to address this more clearly:

Discussion: “This study was conducted using a pot-based experimental setup, which offered a high level of control over irrigation regimes—particularly the treated wastewater and clean water ratios—and allowed for systematic and repeated soil sampling over four consecutive years. However, it is important to emphasize that this approach does not fully replicate field-scale hydrological and biological processes, such as deep percolation, surface runoff, soil heterogeneity, and complex root-soil interactions that naturally occur under open field conditions. These factors can significantly influence the mobility, retention, and bioavailability of heavy metals in soil. Additionally, the study focused solely on the accumulation of heavy metals in the soil compartment, without analyzing their uptake and translocation in plant tissues. This represents a limitation in fully assessing the potential human and ecological risks related to bioaccumulation through the food chain. Therefore, while the findings offer valuable insights under controlled conditions, caution must be exercised when extrapolating these results to real-world agricultural systems. Future research should involve long-term, field-based trials that incorporate both soil and plant analyses to more comprehensively evaluate the environmental sustainability, crop safety, and potential health implications of treated wastewater reuse in agriculture.” (page 15, Line 156-169).

Conclusion: “Although the findings of this study provide valuable insights into the dynamics of heavy metal accumulation under controlled irrigation with treated wastewater, it must be emphasized that the pot-based experimental design does not fully replicate field-scale hydrological and biological conditions. Therefore, the conclusions drawn here should be interpreted with caution, and future research should focus on long-term field experiments to validate these results under real agricultural conditions.” (page 16, Line 192-197).

2- **Comment:** Statistical reporting: The statistical analysis is appropriate but should be reported in greater detail (e.g., include F-values, degrees of freedom, and standard error bars in figures).

**Response:** Thank you for this valuable suggestion. Detailed statistical results are presented in Table 6 to enhance the transparency and clarity of the analysis. In accordance with the reviewer’s recommendation, Figure 6 has been revised to include standard error bars and significance annotations. Different letters now indicate statistically significant differences among treatments (p < 0.05), providing a clearer representation of the results.

3- **Comment:** Lack of plant uptake data: Since only soil data were analyzed, a short discussion on potential bioaccumulation in crops and implications for food safety would improve the ecological relevance of the work.

**Response:** We appreciate the reviewer’s insightful comment regarding the importance of plant uptake data and its implications for food safety. Indeed, understanding the potential transfer of heavy metals from soil to edible plant parts is essential for a comprehensive environmental risk assessment. Although this study focused solely on soil contamination and pollution index evaluations, we agree that including data on heavy metal accumulation in plant tissues would enhance the ecological relevance and public health perspective of the work. In response to this valuable suggestion, we have added a new paragraph in the Levels of heavy metals in soil section (page 12, Line 44-48; 64-74) that outlines relevant findings from the literature regarding bioaccumulation in crops irrigated with wastewater. This paragraph discusses possible health risks, the role of long-term exposure, and emphasizes the need for future studies integrating plant tissue analysis and dietary risk assessments. We believe this addition strengthens the manuscript’s context and supports the need for multidisciplinary approaches in sustainable wastewater reuse practices.

4- **Comment:** Discussion depth: Strengthen the discussion by including mechanistic explanations of metal behavior (adsorption, mobility, pH influence) and by comparing results with recent regional studies, especially from Mediterranean or semi-arid contexts.

**Response:** Thank you for this valuable suggestion. In the revised manuscript, we have significantly expanded the Discussion section by integrating mechanistic insights into the behavior of heavy metals under wastewater irrigation. Specifically, we addressed how key soil properties—such as pH, electrical conductivity (EC), cation exchange capacity (CEC), and organic matter (OM)—influence metal adsorption, mobility, and bioavailability. These explanations are now contextualized with current findings from Mediterranean and semi-arid environments (e.g., Batool et al., 2022; Laptiev et al., 2024; Dogan Demir and Sahin, 2020; Yerli et al., 2025). The revised discussion better links our observed changes in pollution indices with pH- and salinity-driven dynamics and provides a stronger scientific basis for interpreting the behavior of heavy metals in the studied soil systems. We believe this revision considerably enhances the ecological relevance and regional comparability of our findings. (page 8-9, Line 214-226).

5- **Comment:** Figures and tables: Improve figure readability (larger fonts, clear legends, consistent units) and, if possible, include a location map of the study site.

**Response:** Thank you for your constructive feedback. The figure quality has been enhanced to improve readability, including increased resolution and font sizes. All abbreviations used in the figures have been clearly defined in the legend sections for clarity and consistency. Additionally, a location map illustrating the national and regional context of the study area has been added as Figure 1. Map of the study area.

6- **Comment:** Ensure uniform formatting and inclusion of DOIs for all references. Adding more recent studies (2020–2024) would further support the discussion.

**Response:** Thank you for your helpful comment regarding the reference list. In response, we have thoroughly reviewed and revised all references to ensure consistent formatting and included DOI numbers where available. Additionally, to enhance the scientific depth and currency of the discussion, we have incorporated several recent studies published between 2020 and 2024, as recommended. These include: Ahmed et al. (2023), Batool et al. (2022), Ghamarnia et al. (2023), Laptiev et al. (2024), Shi et al. (2020), Tan et al. (2022), Tolossa (2021), Yang et al. (2021), Zhang et al. (2022), Villa et al. (2021), Yerli et al. (2025), Dogan Demir and Sahin (2020), Cakmakci and Sahin (2021), and Slima and Ahmed (2020). We believe these updates significantly strengthen the manuscript's relevance and alignment with current literature.

Thank you once again for your valuable guidance and support. We believe that the changes we have made will meet the reviewers' expectations and improve the manuscript's contribution to the field. We look forward to your feedback.

Sincerely,

Assist. Prof. Dr. Hasan ER
